# Health effects and mediating mechanisms of smart wearable devices on older people: evidence from China

**DOI:** 10.3389/fpubh.2025.1578063

**Published:** 2025-04-25

**Authors:** Enkai Guo, Shiyi He, Lan Zheng

**Affiliations:** ^1^College of P.E, Hunan Normal University, Changsha, China; ^2^Hunan Provincial Research Base for Public Service of Sports, Hunan Normal University, Changsha, China; ^3^Key Laboratory of Physical Fitness and Exercise Rehabilitation of Hunan Province, Hunan Normal University, Changsha, China

**Keywords:** wearable electronic devices, aged, health, exercise, China Longitudinal Aging Social Survey

## Abstract

**Background:**

With the aging of the population, health issues among older people have drawn increasing societal attention. The rapid development of intelligent technology provides innovative solutions to addressing these challenges. This study aims to explore the impact of smart wearable devices on older people’s health outcomes while discussing the mechanism of physical exercise, to provide a new perspective and exercise strategy for maximizing the positive role of smart wearable devices in older adult health management.

**Methods:**

Utilizing data from the China Longitudinal Aging Social Survey (CLASS), this study analyzed 21,881 samples. Descriptive statistics was computed using SPSS, followed by bidirectional fixed effect model analysis and intermediate effect test via Stata.

**Results:**

Bidirectional fixed effect model showed that the use of smart wearable devices could significantly improve the physical health of the older people (*β* = 0.325, *p* < 0.001). The effect was more significant in those aged 70 years or older (*β* = 0.385, *p* < 0.001), those without chronic diseases (*β* = 0.590, *p* < 0.001), and those living with their children (*β* = 0.452, *p* < 0.001). The mediating mechanism test results reported that exercise frequency (*β* = 0.037, *p* < 0.05) and exercise duration (*β* = 0.063, *p* < 0.001) were two positive mediating pathways for smart wearable devices to affect the health of the older adults.

**Conclusion:**

This study reveals the positive impact of smart wearable devices on the physical health level of the older people. To maximize benefits, we propose a dual-path optimization strategy: (1) Develop age-appropriate functions and use methods to improve device accessibility among older adult people, and (2) Generate a personalized exercise prescription for the older people to promote regular physical activity and exercise habits to improve their health level.

## 1 Introduction

Currently, the aging trend of the global population is becoming increasingly conspicuous. Statistical data shows that the world’s population is projected to reach 9.7 billion in 2050 and 11.2 billion in 2100, with an average annual growth rate of 1.18% (1). This demographic shift has precipitated characteristic challenges of an aging society, Such as, particularly evident in the escalating health burdens faced by older people, imposing substantial pressures on both society and families (2, 3). In tandem with the advent of the digital age, smart wearable devices have emerged as a transformative solution within the healthcare domain. By definition, smart wearable devices are a class of intelligent gadgets designed for long-term body-worn usage. Equipped with integrated sensors, advanced data-analysis algorithms, and seamless connectivity features, they enable real-time monitoring and in-depth analysis of users’ health conditions and daily lifestyle patterns (4). Evidently, smart wearable devices have the potential to exert a notable influence on the physical well-being of the older adults, both in terms of continuous health monitoring and providing support for physical activities (5). Against this research landscape, the study is guided by three overarching objectives: (1) To empirically examine whether the utilization of smart wearable devices can effectively improve the physical health of the older adults; (2) To discern the variances in device effectiveness on the health of the older adults across different demographic and health - related conditions, such as different age brackets, chronic diseases status, and cohabitation arrangements; (3) To systematically evaluate the mediating role of physical exercise behavior in the relationship between smart wearable device use and the physical health of the older adults.

Internationally, the research into smart wearable devices for older adult health management has reaped a series of remarkable achievements. In terms of functionality, a large number of studies have been dedicated to enhancing the health monitoring capabilities of these devices to cater the unique needs of the older adults. For instance, efforts have been made to improve the sensitivity of built-in acceleration sensors and increasing the sophistication of internal algorithms, enabling real-time and accurate capture of the older adult’s physical movements (6). Such technological advancements not only provide a proactive approach to fall prevention but also contribute to the overall safety and well-being of the older adults (7). In the sphere of chronic disease management, smart wearable devices have emerged as crucial tools. Take the management of diabetes in the older adults as an example. Specialized smart bracelets have been engineered to facilitate continuous, real-time blood-glucose monitoring (8). This allows doctors to make timely and precise adjustments to treatment plans, including medication dosages (9). Regarding application efficacy, a wealth of evidence-based research has demonstrated the positive influence of smart wearable devices in older adult health management (10). A long-term follow-up study shows that smart wearable devices can significantly enhance users’ awareness of their own physical conditions, thereby continuously motivating them to increase their physical activity levels by providing exercise reminders and personalized health advice (11). Thus, smart wearable devices hold great promise in promoting healthy aging. At the same time, numerous domestic studies in China have indicated that the multifarious functions of smart wearable devices, including but not limited to heart rate monitoring, breath tracking, and skin humidity sensing, endow them with the capacity to conduct a more all-encompassing assessment of the older adult’s health conditions (12). This comprehensive assessment capability represents a significant breakthrough for the wider penetration of smart wearable devices among the older adults demographic (13). In summary, the utilization of smart wearable devices is intricately associated with the health conditions of the older adults, and exercise also plays a role in this context. Consequently, this study proposes the following hypotheses:

*H*1: Employing smart wearable devices can notably enhance the physical health of elder adults.

*H*2: Through the mediating effect of exercise engagement, smart wearable devices can further boost the physical health of the older adult population.

Simultaneously, certain studies have indicated that due to the heterogeneous nature of the older adult population, there exists a substantial disparity in health-related costs, which can influence the health-promoting effects of smart wearable devices. Factors such as age (14), health conditions (15), and living arrangements will all have an impact on the health-related costs incurred by the older adults, resulting in different levels of health efficacy derived from smart wearable devices. Therefore, in light of the above-mentioned characteristics of the older adults, this study puts forward the following research hypotheses:

*H*3a: Older individuals are more inclined to enhance their physical health by using smart wearable devices.

*H*3b: Older adult people with chronic diseases have a higher likelihood of improving their physical health through the utilization of smart wearable devices.

*H*3c: Older adult individuals co-living with their children are more likely to improve their physical health by utilizing smart wearable devices.

## 2 Materials and methods

### 2.1 Data sources

This study draws upon data from the China Longitudinal Aging Social Survey (CLASS) database (available at http://class.ruc.edu.cn/). CLASS is a database jointly developed and curated by the Institute of Gerontology and the Institute of Population and Development Studies at Renmin University of China. Initiated in 2014, it has been subject to biennial tracking, employing a stratified multi-stage probability sampling method. This sampling approach ensures broad representativeness, covering 30 provinces (including municipalities and districts), over 400 village-level units, and more than 11,000 older adult individuals aged 60 and above (4). Since 2018, the survey has incorporated questions related to smart devices, providing substantial support for exploring the relationship between relevant smart devices and the health of the older adults in the digital age. The inclusion of such questions enables in-depth analysis of how smart devices are integrated into the lives of the older adults and their subsequent impact on health-related outcomes.

Therefore, leveraging the survey data from 2018 and 2020, this study constructs a research panel focused on the influence of smart wearable devices on the physical health of the older people. Firstly, this study employs a two-way fixed effects model to determine whether the use of smart wearable devices can enhance the health status of the older adults, and to clarify the different effects of such influence in terms of age, health conditions, and living arrangement. Secondly, this study utilizes a mediation effect model, through stepwise regression analysis, to explore the mediating role of exercise behavior, aiming to further clarify the pathway through which smart wearable devices promote the health of the older adults. After excluding samples with missing values, a total of 21,881 samples were included for analysis, and the StataMP 16 software was employed for the statistical analysis.

### 2.2 Variable selection

#### 2.2.1 Dependent variable

In the present study, self-rated health status (16) was chosen as the dependent variable (denoted as “Physical_H “) to evaluate the physical health conditions of the older adults. Participants were asked, “How would you rate your overall health?” Their responses were measured on a 5-point Likert scale, ranging from “very good” (assigned a value of 5) to “very poor” (assigned a value of 1), as presented in Table 1.

**Table 1:** Descriptions of variables.

Variable	Description	Coding values
Dependent variable		
Physical_H	Self-rated physical health of the older people	Measured on a scale of 5 (very good) to 1 (very poor)
BMI	Body Mass Index = weight (kg) / height^2^ (m)	A BMI value of 18.5–24 is normal, and the value is 1. BMI higher than 24 is overweight, lower than 18.5 is thin, both are abnormal, assigned a value of 0
Independent variable		
Device	Whether to own and use smart wristbands and smart watches	Yes = 1, no = 0
Mechanism variable		
Exercise_F	Frequency of participation in physical activity	Exercise twice or more per week is assigned a value of 1, otherwise 0
Exercise_D	The length of time each time you participate in physical exercise	Less than 30 min = 1; 30–59 min = 2; 60–120 min = 3; Higher than 120 min = 4
Controlled variable		
Gender	Gender	Male = 1, female = 0
Age	Age	Measured in years
Education	Educational level	Illiteracy = 1; Private school/Literacy class = 2; Primary school = 3; Junior high = 4; High school/technical secondary school = 5; Junior college = 6; Bachelor’s degree or above = 7
Marriage	Martial status: whether you are married and have a spouse?	Yes = 1, no = 0, “no” including widowhood, divorce, unmarried, etc
Urban	Whether you live in an urban area?	Yes = 1, no = 0
Solitude	Live alone or not	Yes = 1, no = 0
Economic	Personal annual income is divided into high income and low income groups with 20,000 yuan as the boundary (21)	High = 1, low = 0
Offspring	Number of children	
Pension	Do you have pension insurance? It includes basic old-age insurance for enterprise employees (basic pension for urban employees), old-age insurance for government agencies and public institutions (retirement pension for government agencies and public institutions), and basic old-age insurance for urban and rural residents	Yes = 1, no = 0
LnGDP	The economic development level of the city is expressed as the logarithm of the regional GDP per capita	

Furthermore, in the robustness test segment, the Body Mass Index (BMI) was incorporated into this study. By calculating the BMI, which is determined by dividing an individual’s weight in kilograms by the square of their height in meters, this study aimed to objectively evaluate the physical health level of the older adults. Specifically, the BMI index was utilized to identify whether the older adults were overweight or underweight. This objective measure complements the self-rated health status, offering a more comprehensive understanding of the physical health of older adults. The use of the BMI index allows for a more quantifiable and standardized assessment, thereby enhancing the rigor and reliability of the research findings regarding the physical health of the older adults.

#### 2.2.2 Independent variable

Smart wristbands and smart watches, representing a category of consumer-health products within the broad spectrum of smart wearable devices, are equipped with functions such as information monitoring and feedback, data positioning, and social interaction capabilities. These multifaceted functions furnish crucial technical support for the older adults to adjust and modify their health-management strategies, thereby exerting a significant influence on the health status of the older adult population. In this study, in alignment with the question “Do you own a smart wristband/watch” incorporated in the CLASS questionnaire, an attempt was made to gage the impact of smart wearable devices on the physical health of the older adults. A virtual variable, denoted as “Device,” was employed for this purpose. A value of 1 assigned to the variable “Device” indicates that the older adult individual owns a smart wristband or watch, while a value of 0 signifies the absence of such a device.

#### 2.2.3 Controlled variables

Building upon existing literature, this study incorporates several control variables: Gender, Age, Education, marital status (Marriage), type of residence (Urban), Solitude, annual personal income (Economic), number of children (Offspring), Pension, and the economic conditions of the locality (LnGDP). The definitions and measurement methodologies of each control variable are detailed in Table 1. These variables are measured from three dimensions. Firstly, individual basic characteristics include gender, age, education level, and marital status. Secondly, individual socioeconomic status comprises both social and economic characteristics. Social characteristics include residence type, solitude, and number of children, while economic characteristics involve personal annual income and pension. Environmental status is represented by local economic conditions, measured using the logarithm of local GDP. The selection of these control variables considers both macro and micro dimensions, minimizing measurement error to the greatest extent possible.

#### 2.2.4 Mechanism variables

Previous research has indicated that sports participation behavior is a crucial factor influencing the health status of the older adults. Consequently, this study employed exercise frequency (Exercise_F) and exercise duration (Exercise_D) as mediating variables to explore the impact of smart wearable devices on the physical health of the older adults. Exercise frequency refers to the number of times older people participate in exercise per week, while exercise duration pertains to the length of each exercise session undertaken by the older people.

### 2.3 Model construction

To rigorously examine the impact of smart wearable devices on the physical health of the older adults, considering the characteristics of the dependent variables, the ordered logit method was employed to construct the baseline regression model for this study, denoted as Equation 1. This approach is particularly suitable for handling dependent variables that are ordinal in nature, such as the self-rated health status of the older adults, which typically falls on an ordered scale (e.g., very good, good, fair, poor, very poor).


(1) $$Y_{\mathrm{it}}=\beta_{0}+\beta_{1}X_{\mathrm{it}}+\sum \beta_{k}z_{\mathrm{it}}+\varepsilon_{\mathrm{it}}$$

Y is the explained variable, which represents the subjective physical health status of the i-th older person; t is the year.

X is the explanatory variable, indicating whether smart wearable devices are used.

Z includes control variables, such as gender, age, marital status, education, etc.

*ε* is a random disturbance term following the Logistic distribution.

β_0_ is the intercept term, while β_1_ and β_k_ are parameters to be estimated.

In the context of action path analysis, this study delved deeper into the impact of smart wearable devices on the health of the older adults using a mediating effect model. The methodological approach primarily involves the following steps:

Step A: Construct a regression equation that quantifies the impact of smart wearable devices on the health of the older adults, denoted as Equation 2. This equation serves as the foundation for understanding the direct relationship between the use of smart wearable devices and the health status of the older adults.

Step B: Upon establishing that the regression coefficient of smart wearable devices in Equation 2 is statistically significant, the subsequent step is to construct two additional regression equations. The first, Equation 3, examines the influence of smart wearable devices on the mediating variables. The second, Equation 4, analyzes the combined influence of smart wearable devices and the mediating variables on the physical health of the older adults. By estimating these equations, a comprehensive test can be conducted to determine whether a mediating effect exists.

If the coefficient of the mediating variable in Equation 4 is significant and the coefficient of the smart wearable device variable changes (either in magnitude or significance) compared to Equation 2, it provides evidence of a mediating effect, thus shedding light on the underlying mechanisms through which smart wearable devices impact the health of the older adults. Based on this framework, the specific model is outlined as follows:


(2) $$Y_{\mathrm{it}}=\alpha_{0}+\alpha_{1}X_{\mathrm{it}}+\sum \alpha_{k}z_{\mathrm{it}}+\varepsilon_{\mathrm{it}}$$

(3) $$M_{\mathrm{it}}=\beta_{0}+\beta_{1}X_{\mathrm{it}}+\sum \beta_{k}z_{\mathrm{it}}+\varepsilon_{\mathrm{it}}$$

(4) $$Y_{\mathrm{it}}=\gamma_{0}+\gamma_{1}X_{\mathrm{it}}+\gamma_{2}M_{\mathrm{it}}+\sum \gamma_{k}z_{\mathrm{it}}+\varepsilon_{\mathrm{it}}$$

In the above equation:

M represents the mediating variable, including exercise frequency and duration. While other variables in the formula are consistent with the previous definitions.

This study focuses on estimating the coefficients α_1_/β_1_/γ_1_/γ_2_.

## 3 Results

### 3.1 Descriptive analysis

Based on the descriptive statistical analysis of the categorical variables presented in Table 2, it becomes evident that nearly 50% of the respondents rated their physical health as either good or very good. This data provides initial insights into the self-perception of health among the older adults population under investigation. A mere 6.03% of the older adults within the sample possess smart wearable devices. This figure vividly demonstrates that the adoption rate of smart wearable devices among the older adults in China remains at a relatively low level and is in urgent need of improvement. This low penetration rate may be attributed to various factors, such as technological literacy barriers among the older adults (17), concerns regarding device usability and privacy (18), as well as the relatively high cost of these devices.

**Table 2:** Descriptive statistics of categorical variables.

Variable	Description	Frequency	Percent
Physical_H	Very Good	1,685	7.70%
	Good	8,464	38.68%
	Fair	8,237	37.64%
	Poor	2,973	13.59%
	Very poor	522	2.39%
BMI	Normal	14,562	66.55%
	Abnormal	7,319	33.45%
Device	Yes	1,320	6.03%
	No	20,561	93.97%
Gender	Male	11,012	50.33%
	Female	10,869	49.67%
Education	Illiteracy	5,318	24.30%
	Private school/Literacy class	904	4.13%
	Primary school	8,052	36.80%
	Junior high	5,310	24.27%
	High school/technical secondary school	1794	8.20%
	Junior college	421	1.92%
	Bachelor’s degree or above	82	0.37%
Marriage	Yes	15,919	72.75%
	No	5,962	27.25%
Urban	Yes	12,454	56.92%
	No	9,427	43.08%
Solitude	Yes	2,455	11.22%
	No	19,426	88.78%
Economic	High	2,392	10.93%
	Low	19,489	89.07%
Pension	Yes	16,876	77.13%
	No	5,005	22.87%
Exercise_F	Exercise twice or more per week	4,063	36.80%
	Exercise once a week or less	6,977	63.20%
Exercise_D	Less than 30 min	8,280	75.00%
	30–59 min	1707	15.46%
	60–120 min	986	8.93%
	Higher than 120 min = 4	67	0.61%

As depicted in Table 2, the sample exhibits an approximately equal gender distribution, with males and females represented in almost equal proportions. Fewer than 50% of the older adult participants have attained secondary or higher education. The majority of the older adults are married and have spouses. The proportion of older adult individuals residing in urban areas constitutes 56.92% of the sample. An overwhelming majority of the older adults do not live alone. Moreover, over 88.78% of the older adults have an annual income of less than 20,000 yuan, and 77.13% of them receive a pension. The descriptive statistics for continuous variables in Table 3 reveal that the mean age of the 21,881 participants is 71 years.

**Table 3:** Descriptive statistics of continuous variables.

Variable	Observation	M	SD	Min	Max
Age	21,881	71.402	6.936	52	108
Offspring	21,881	2.513	1.367	0	10
LnGDP	21,881	11.123	0.397	10.353	12.013

From the perspective of mediating variables, the exercise frequency of older adult people is once a week or less, and most exercise sessions last for less than 30 min. This implies that the physical activity levels of the older adults in the sample are relatively low, which could potentially impact their overall health and well-being. Understanding these patterns of exercise behavior is essential for further analyzing how smart wearable devices could potentially encourage more active lifestyles among the older adults.

### 3.2 Basic regression

In this research, the Hausman test is employed to discern between the fixed-effects model and the random-effects model. In Table 4, Columns (1) and (2) respectively present the estimations derived from the fixed-effects approach and the random-effects approach. The outcomes of the Hausman test suggest that the fixed-effects model is more appropriate for this study. Consequently, all subsequent empirical analyses are conducted using the fixed-effects model.

**Table 4:** Baseline regression results.

Variables	Physical_H			
	(1) FE	(2) RE	(3) FE	(4) FE
Device	0.331^***^ (0.030)	0.309^***^ (0.022)	0.342^***^ (0.031)	0.325^***^ (0.031)
Gender	0.130 (0.160)	0.060^***^ (0.015)		0.057^***^ (0.014)
Age	−0.003 (0.005)	−0.015^***^ (0.001)		−0.016^***^ (0.001)
Education	0.045 (0.039)	0.036^***^ (0.006)		0.046^***^ (0.007)
Marriage	0.140^***^ (0.047)	0.153^***^ (0.018)		0.152^***^ (0.018)
Urban	0.126^***^ (0.031)	0.040^***^ (0.015)		0.054^***^ (0.017)
Solitude	0.202^***^ (0.037)	0.128^***^ (0.022)		0.134^***^ (0.026)
Economic	−0.093^***^ (0.024)	−0.074^***^ (0.018)		−0.051^**^ (0.023)
Offspring	−1.036 (0.752)	−0.020^***^ (0.006)		−0.012^*^ (0.006)
Pension	−0.046^*^ (0.024)	−0.014 (0.015)		−0.043^**^ (0.019)
LnGDP	−0.196^***^ (0.074)	−0.079^***^ (0.019)		−0.148^**^ (0.073)
_cons	8.006^***^ (2.007)	5.053^***^ (0.214)	3.205^***^ (0.030)	5.728^***^ (0.871)
R^2^	0.014	0.045	0.069	0.106
F	18.81^***^	877.35^***^	1227.47^***^	2022.36^***^
N	21,881	21,881	21,881	21,881
Hausman Test	29.07^***^			
City			Controlled	Controlled
Year			Controlled	Controlled

(1) *p < 0.1. **p < 0.05. ***p < 0.01. (2) In column (3) & (4), robust standard error for t-statistics in parentheses.

Columns (3) and (4) report the model estimations based on the fixed-effects approach. After incorporating control variables and fixed effects, the coefficient of the variable “Device” in relation to the variable “Physical_H” is found to be 0.325, and this coefficient is statistically significant at the 1% level. This finding implies that, after controlling for various influencing variables, the adoption of smart wearable devices can significantly enhance the health level of the older adults. Thus, H1 is supported by the empirical evidence.

Moreover, the estimated results of the relevant control variables are generally consistent with prior expectations. Older men tend to rate their own physical health more positively than older women (*β* = 0.057, *p* < 0.01). There is a negative correlation between age and health status (*β* = −0.016, *p* < 0.01), a finding consistent with the well-documented physiological phenomenon that as individuals age, the functionality of various bodily systems gradually deteriorates. Education level is positively correlated with health (*β* = 0.046, *p* < 0.01), suggesting that a higher level of education equips the older adults with better health awareness, enabling them to adopt healthier lifestyles and preventive measures. Urban residents generally exhibit better health status compared to their rural counterparts (*β* = 0.054, *p* < 0.01), a disparity likely attributed to the more advanced medical resources and superior material living conditions available in urban areas. Contrary to some intuitive assumptions, older individuals with lower annual personal incomes (*β* = −0.051, *p* < 0.05), those without pension support (*β* = −0.043, *p* < 0.05), and those residing in economically less-developed areas (*β* = −0.148, *p* < 0.05) often report relatively better physical conditions. One possible explanation for this phenomenon is that these individuals may engage in more physical activities to meet their basic living needs. Over the long term, regular physical activity can contribute to maintaining good physical health and providing the necessary support for their well-being.

### 3.3 Robustness test

To ensure the precision and reliability of the research findings, a series of robustness tests were conducted. These tests involved altering the measurement approach of the dependent variable, adjusting the fixed-effect specifications, and changing the measurement model.

**Figure 1:**
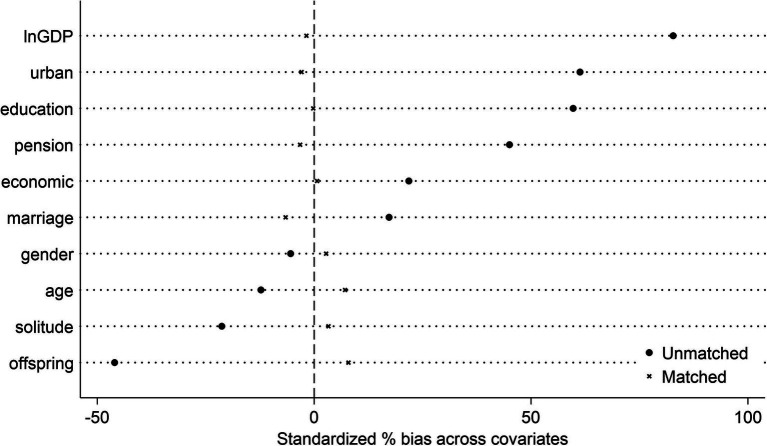
Graph of the standardized deviation of each variable.

First, the Body Mass Index (BMI) was employed as the dependent variable, serving as an objective and widely-recognized metric of physical health among the older adults. Second, to account for potential omitted variable bias across different macro-regions, regional fixed effects were introduced for the eastern, central, and western regions. These regional disparities, stemming from variations in economic development, healthcare resources, and cultural factors, can significantly impact the relationship between smart wearable device usage and older adult health outcomes. Lastly, to mitigate the selection bias from observable variables, Propensity Score Matching (PSM) method was applied to pre-process the samples (4). Smart wearable device usage was treated as a binary treatment variable, and year-by-year nearest-neighbor matching was carried out based on a set of covariates. Unmatched samples were excluded, and fixed-effect regression analysis was subsequently performed. Figure 1 depicts the standardized deviations of each variable before and after matching. For most variables, the standardized deviation decreased post-matching, suggesting that PSM method effectively balanced covariates between treatment and control groups, enhancing sample comparability and reducing potential selection bias.

The outcomes of these three robustness tests are presented in Table 5. Across all these tests, the core conclusion of this paper remains consistent, reinforcing the reliability and generalizability of the research findings.

**Table 5:** Robustness test results.

Variables	Change dependent variable	Change fixed effect	Change measurement model
	(1) BMI	(2)	(3) PSM
Device	0.066^***^(0.017)	0.047^***^(0.016)	0.307^***^(0.038)
Controlled variables	Controlled	Controlled	Controlled
City	Controlled	No	Controlled
Year	Controlled	Controlled	Controlled
District	No	Controlled	No
Pseudo R^2^	0.025	0.025	0.283
N	21,881	21,881	21,324

(1) ***p < 0.01. (2) Robust standard error for t-statistics in parentheses.

### 3.4 Heterogeneity analysis

Prior research has demonstrated that the influence of smart wearable devices on older adults physical health varies by factors such as age, chronic disease status, and living arrangements (specifically, whether they co-reside with their children). Building on these insights, this study undertook a heterogeneity analysis to explore the differential effects across these subgroups.

As presented in Table 6, the utilization of smart wearable devices exerts a more pronounced and statistically significant impact on the physical health of certain older adult subgroups: Individuals aged 70 years and above (*β* = 0.385, *p* < 0.01), those without chronic diseases (*β* = 0.590, *p* < 0.01), and those living with their children (*β* = 0.452, *p* < 0.01).

**Table 6:** Heterogeneity test results.

Variables	Age		Chronic disease		Cohabitation with children	
	(1) 60–69 years old	(2) Age 70 and older	(3) No	(4) Yes	(5) No	(6) Yes
Device	0.270^***^ (0.040)	0.385^***^ (0.045)	0.590^***^ (0.121)	0.322^***^ (0.033)	0.291^***^ (0.040)	0.452^***^ (0.054)
Controlled variables	Controlled	Controlled	Controlled	Controlled	Controlled	Controlled
City	Controlled	Controlled	Controlled	Controlled	Controlled	Controlled
Year	Controlled	Controlled	Controlled	Controlled	Controlled	Controlled
Pseudo R^2^	0.088	0.107	0.153	0.115	0.105	0.118
N	10,273	11,608	5,604	16,277	14,388	7,493

(1) ***p < 0.01. (2) Robust standard error for t-statistics in parentheses.

These findings support hypotheses H3a, H3b, and H3c. H3a, which posits that older people are more likely to improve their physical health through the use of smart wearable devices, is validated by the significant impact observed among the 70-plus age group. H3b, suggesting that older adult people with chronic diseases are more likely to benefit, is nuanced by the finding that those without chronic diseases also gain substantial health improvements, woffering a more complex perspective on the device’s effectiveness. H3c, stating that older adult people living with their children are more likely to see health improvements, is confirmed by the results indicating a stronger effect in this subgroup. These findings deepens our understanding of how smart wearable devices influence the physical health of the older adults, and highlights the importance of tailoring health interventions to specific demographic and health-related factors.

### 3.5 The mechanism of smart wearable devices on the physical health of the older people

The utilization of smart wearable devices has the potential to enhance the enthusiasm and scientific approach of the older adults toward physical exercise, which is directly correlated with their physical health. Having established the devices’ positive impact on older adult physical health, this study further explores the underlying mechanism through the lens of physical exercise behavior.

In this research, step-wise regression was employed to test the mediating effect of exercise frequency, followed by Bootstrap validation with 500 random repeated samplings. As shown in Table 7. By comparing the magnitudes of the total effect and the direct effect, it was discovered that the direct effect (*β* = 0.358, *p* < 0.01) is lower than the total effect (*β* = 0.362, *p* < 0.01), with the mediating effect stands at 1.11%. This indicates that exercise frequency plays partially mediates the relationship between smart wearable device usage and older adult physical health, supporting Hypothesis H2.

**Table 7:** Mechanism of exercise behavior.

Variables	Physical_H	Exercise_F	Physical_H	Exercise_D	Physical_H
	(1)	(2)	(3)	(4)	(5)
Device	0.362^***^(0.031)	0.120^***^(0.014)	0.358^***^(0.031)	0.053^**^(0.025)	0.359^***^(0.031)
Exercise_F			0.037^**^(0.019)		
Exercise_D					0.063^***^(0.013)
_cons	6.505^***^(1.166)	−2.235^***^(0.514)	6.587^***^(1.169)	−7.061^***^(0.412)	6.948^***^(1.173)
Controlled variables	Controlled	Controlled	Controlled	Controlled	Controlled
City	Controlled	Controlled	Controlled	Controlled	Controlled
Year	Controlled	Controlled	Controlled	Controlled	Controlled
R^2^	0.129	0.186	0.129	0.261	0.130
N	11,040	11,040	11,040	11,040	11,040
Direct effect	0.358^***^(0.031)			0.359^***^(0.031)	
Indirect effect	0.004^*^(0.002)			0.003^**^(0.002)	
Total Effect	0.362^***^(0.031)			0.362^***^(0.031)	

(1) *p < 0.1. **p < 0.05. ***p < 0.01. (2) Robust standard error for t-statistics in parentheses.

To further measure the role physical exercise behavior, exercise duration was analyzed as an additional mediating variable. As indicated in Table 7, its mediating effect is also significant, accounting for 0.83% of the total effect, further validating Hypothesis H2.

**Figure 2:**
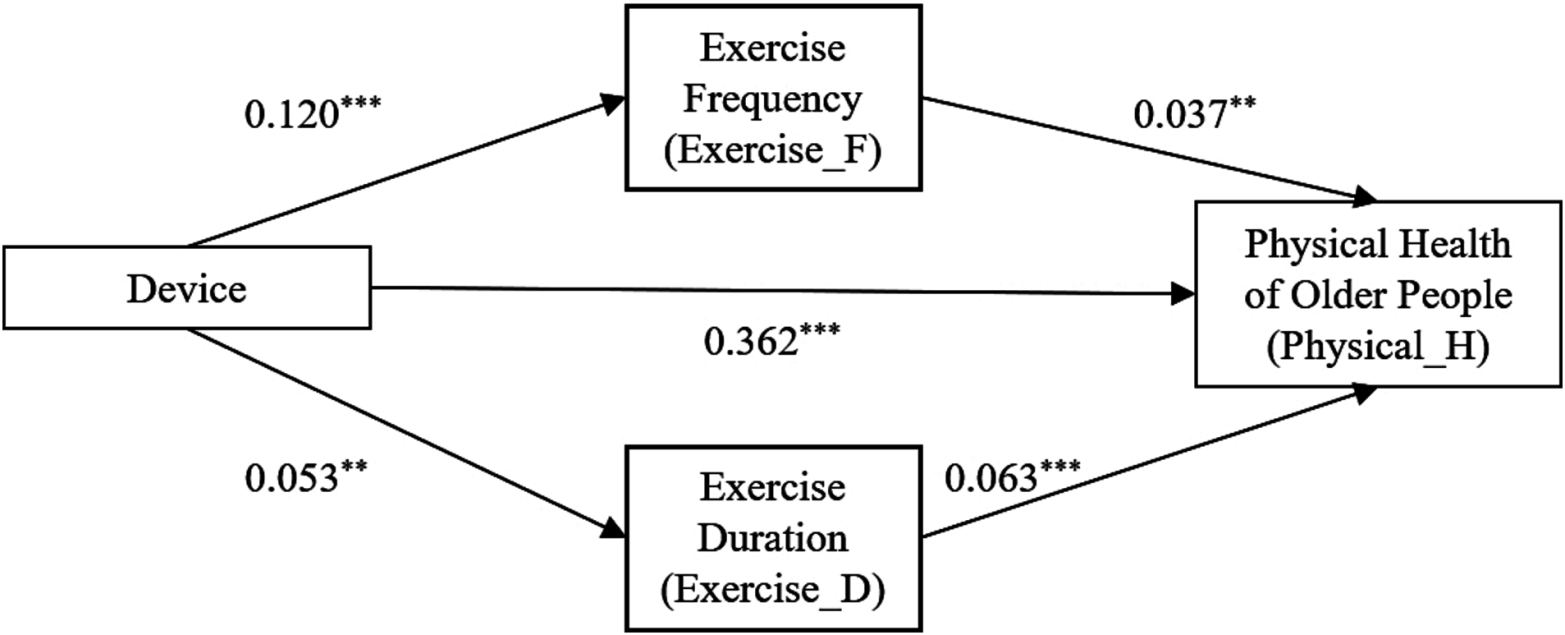
Pathways of the impact of smart wearable devices on the physical health of the older people.

In conclusion, Figure 2 illustrates the mediation model, confirming that physical exercise behavior partially explains the link between smart wearable device usage and older adult physical health. These findings highlight the role of exercise as a key pathway through which wearable technology influences health outcomes in aging populations.

## 4 Discussion

### 4.1 Main findings

With the arrival of the digital era, bridging the digital gap for the older adult population and enabling them to benefits from intelligent technologies, particularly digital tools, has emerged as a crucial approach to proactively addressing the challenges posed by population aging. Against this backdrop, this study draws on the 2018 and 2020 China Longitudinal Aging Social Survey (CLASS) data to comprehensively elucidate the impact of smart wearable devices on older adult health. The primary objective is to provide empirical evidence of their positive effects. Findings indicate that the utilization of smart wearable devices significantly improves older adult health, aligning with previous research (19).

Heterogeneity analysis reveals that this impact varies significantly across different dimensions, namely age, disease status, and living arrangements. First, while smart wearables benefit both younger and older adult groups, the effect is more pronounced among those aged 70 and above. As health declines with age, individuals over 70 have greater potential for improvement, making smart wearables particularly beneficial. Second, the devices enhance older adult physical health outcomes regardless of chronic disease status, but the effect is more notable among those without chronic conditions. Finally, smart wearables positively influence older adult physical health across different living arrangements, yet the effect is more prominent for those living with their children. This is closely related to the inherent characteristics of smart wearable devices.

The results of the mediation analysis indicate that sports participation can serve as a mediating factor, which is in line with the outcomes of previous research (20). First, regarding exercise frequency, smart wearable devices help older adult users increase their exercise frequency. By cultivating regular and healthy exercise habits, the health conditions of the older adults can be remarkably enhanced. This discovery implies that smart wearable devices can efficiently boost the exercise participation rate among the older adults and support the long-term development of good exercise habits. Future device updates of smart wearable devices could focus on real-time reminders for the older adults to encourage consistent physical activity (21). Second, exercise duration plays a crucial role in maximizing the health benefits of smart wearable devices. With the advancement and popularization of intelligent and digital technologies, older adult individuals are increasingly integrating smart devices into their daily lives (22). These devices not only provide exercise-related knowledge and motivation but also help users avoid common mistakes, ensuring a more scientific and sustainable fitness routine (17). As a result, the older adults can access scientifically grounded fitness guidance, track exercise duration in real-time, and maintain medium-to-high-intensity activity according to the device’s reminders, ultimately leading to better health outcomes.

### 4.2 Comparison with evidence

In the realm of control variables, two rather peculiar findings have emerged. The first is that the older adults who live alone exhibit better health conditions, which challenges conventional assumptions. Traditionally, co-residing with children is believed to provide both spiritual and financial support, contributing to older adult well-being (23, 24). However, many older adult individuals today prefer living alone, allowing them to structure their daily routines according to their own preferences. They maintain regular social interactions with their children and others while enjoying ample personal time, free from caregiving responsibilities for grandchildren (25). This autonomy supports better mental health, stable routines, and consistent exercise habits. The second finding is that the older adults with poorer economic conditions tend to have better health, contradicting previous research indicating that economic stability is a prerequisite for the older adult well-being (26, 27). Nevertheless, in China, economic factors no longer appear to be the decisive factor for older adult health. With the success of nationwide poverty alleviation efforts, residents’ basic living needs are largely met. On this basis, wealthier older adult individuals often seek to maintain their health through passive methods such as consuming health products and receiving medical treatments (28). In contrast, those with limited economic resources tend to adopt active means such as physical activities to reduce healthcare-related expenses. Evidently, active health-promotion methods like exercise are more beneficial to the older adults than passive approaches such as relying on health supplements.

In terms of heterogeneity analysis, first, for the older adults aged 70 and older, smart wearable devices provide real-time health monitoring, enabling early detection of potential health problems and timely medical intervention. This feedback mechanism is of great significance for those with declining physical functions, as it can effectively prevent the exacerbation of diseases. Second, chronic conditions like diabetes and hypertension are highly prevalent among the older adults. However, those free from chronic diseases typically enjoy better physical fitness due to their long-standing healthy lifestyle. Their regular diet, exercise, and social habits sustains their good health. Smart wearable devices further support this by allowing them to quantify daily exercise intensity and monitor vital data such as heart rate, blood oxygen saturation, and step count, thereby further maintaining physical well-being (29). Finally, older people who live together with their children benefit from assistance in using smart wearable devices and engaging in physical exercises. Currently, most wearables are not specifically designed for older adult users, often featuring small fonts, complex functions, and overly sensitive interfaces, which make it difficult for the older adults to operate (13). However, with their children’s guidance, older adult users can gradually navigate these functions with minimum trial and error, maximizing their health benefits.

### 4.3 Policy implication

From the conclusions of this study, the following policy implications can be summarized. First, it is crucial to focus on the cross-scene and cross-demographic application and promotion of smart wearable devices. In economically underdeveloped, remote, and rural areas, subsidy policies should be formulated to help older adult individuals afford these devices. This can prevent digital exclusion caused by structural disadvantages and help bridge the health gap among the older adults of varying socioeconomic statuses.

Second, the research and development of age-friendly functions of smart wearable devices need to be strengthened. Considering the characteristics of the older adults, devices should feature large, easy-to-read fonts, simple yet comprehensive functions, and easy-to-operate interfaces should be developed. This will effectively meet the consumption demands of the older adults.

Third, smart wearable device manufacturers should be encouraged to integrate personalized exercise programs for the older adults. By considering factors such as age, gender, sports preferences, and disease status, personalized exercise programs can be designed. This not only fosters greater participation in sports and exercise but also disseminates sports and health knowledge, helping the older adults develop regular exercise habits.

### 4.4 Strengths

This study significantly advances knowledge in smart wearable devices and older adults physical health. First, it uncovers the mediating role of exercise participation between smart wearables and older adults’ health. By dissecting the underlying mechanism, it highlights the devices’ potential to encourage exercise and promote well-being, emphasizing the need to boost adoption and bridge the digital divide among the older adults. Second, using two-year CLASS data for empirical analysis offers a model for future large-sample studies. The longitudinal approach helps explore long-term effects on various health aspects, and the large-scale dataset enhances generalizability. Third, analyzing older adult heterogeneity by age, disease, and living arrangements provides a nuanced view. This enables the development of personalized health strategies and helps healthcare providers make tailored recommendations.

### 4.5 Limitations

This study has two primary limitations. First, the independent variable is restricted to a binary measure of smart wearable device usage. Future research should delve into the diverse types and functions of these devices to better understand their specific impacts on older adult’s well-being. Second, this study centers solely on the physical health of the older adults. In subsequent investigations, relevant variables related to mental and social health should be incorporated to offer a more holistic perspective on aging. Expanding the scope in this way would provide a more complete understanding of how smart wearables contribute to overall well-being in later life.

## 5 Conclusion

Smart technology has revolutionized health services for the older adults, presenting substantial opportunities to elevate the health standards of the older adult population and foster their seamless integration into the social milieu. Leveraging the micro-survey data from the China Longitudinal Aging Social Survey (CLASS) in 2018 and 2020, this study empirically examined the impact of smart wearable devices on older adult health and its underlying intermediary mechanisms through the employment of a two-way fixed-effects model. The findings reveal that the utilization of smart wearable devices exerted a pronounced and statistically significant impact on older adult physical health. This conclusion was further validated by a series of robustness tests, ensuring its reliability and stability. Heterogeneity analysis further indicate that the health-promoting effects of smart wearable devices on the older adults varied significantly across different dimensions. These dimensions included distinct age groups, the presence or absence of chronic diseases, and living arrangements (whether living with children or not). Notably, the improvement in physical health was even more significant for those aged 70 years and older who did not have chronic diseases and who lived with their children. The mediating analysis demonstrated that the use of smart wearable devices could enhance the health of the older adults primarily by augmenting the frequency and duration of physical exercise. Evidently, exercise behavior played a significant mediating role in this process, highlighting the importance of promoting physical activity among the older adults through the application of smart wearable technologies.

## Data Availability

Publicly available datasets were analyzed in this study. This data can be found at: China Longitudinal Aging Social Survey (CLASS), http://class.ruc.edu.cn/.
